# Purification and characterization of recombinant human renin for X-ray crystallization studies

**DOI:** 10.1186/1471-2091-9-19

**Published:** 2008-06-26

**Authors:** Zhongren Wu, Maria G Cappiello, Boyd B Scott, Yuri Bukhtiyarov, Gerard M McGeehan

**Affiliations:** 1 Vitae Pharmaceuticals Inc., Discovery Biology, Fort Washington, PA, 19034, USA

## Abstract

**Background:**

The renin-angiotensin-aldosterone system (RAS) cascade is a major target for the clinical management of hypertension. Although inhibitors of various components of this cascade have been developed successfully, development of renin inhibitors has proven to be problematic. The development of these inhibitors has been hindered by poor bioavailability and complex synthesis. However, despite the challenges of designing renin inhibitors, the enzyme remains a promising target for the development of novel treatments for hypertension. X-ray crystallographic data could greatly assist the design and development of these inhibitors. Here we describe the purification and characterization of recombinant human renin for x-ray crystallization studies.

**Results:**

A cDNA encoding the full length of native human preprorenin (406 amino acid residues) was introduced into the HEK-293 cell line. A clonal cell line expressing prorenin was generated and grown under serum free conditions in a hollow fiber bioreactor. Prorenin was constitutively secreted and purified directly from the conditioned medium. Concanavalin A chromatography effectively enriched and purified prorenin to 90% homogeneity in a single step. Prorenin was converted to active renin by trypsin digestion to remove the propeptide. Active renin was further purified using a cation exchange column followed by a gel filtration column. Biochemical characterization of the recombinant enzyme showed both binding and catalytic properties were essentially identical to previously reported activities for purified renin. Crystals were grown using this material in our X-ray structure studies, and high resolution diffraction was obtained.

**Conclusion:**

This present work describes a simple and efficient method for the generation and purification of active human renin. The protein is highly pure and is suitable for supporting structural biology efforts.

## Background

The renin-angiotensin system (RAS) is a hormone system that regulates blood pressure and extracellular volume in the body. The RAS sequentially processes angiotensinogen to angiotensin II (Ang II), a peptide hormone that is a potent vasoconstrictor. Inhibition of RAS components has been used successfully in the treatment of hypertension, heart failure and end organ damage. Renin catalyzes the first and rate-limiting step of the RAS cascade and renin is specific for angiotensinogen. Blockade of Ang II production by direct inhibition of renin has long been a therapeutic goal. Early renin inhibitors, such as enalkiren and remikiren, were effective in blood pressure lowering. However, due to poor oral bioavailability, duration of action, and high costs of synthesis, these early peptidomimetic inhibitors never progressed to pivotal clinical studies [1]. Continued clinical interest in renin has led to the recent approval of the first renin inhibitor, aliskiren (Tekturna™), a non-peptidic inhibitor of renin, which has generated an interest in finding newer renin inhibitors with improved profiles.

Renin is a member of the aspartic acid protease family. The human renin gene encodes for a protein consisting of 406 amino acids. It is proteolytically processed and secreted as a 384 residue zymogen, prorenin, which contains a 46 amino acid propeptide. The propeptide serves as an autoinhibitory domain that controls the enzyme activity. The pro segment must be removed to generate mature, fully active renin. Prorenin is synthesized principally by juxtaglomerular cells in the wall of the afferent arteriole of the kidney and is released into the bloodstream.

Renin was first purified from animal tissues, including submaxillary glands and kidneys. Yields were generally very low. For instance, Yokosawa et al [2] obtained only 440 μg of pure renin from 22 kg of non-cancerous autopsied human kidneys. Using recombinant techniques, renin has been expressed and purified from eukaryotic cell lines including CHO cells[3,4], mouse L-929 cells, sf9 insect cells [5] and DAMP cells [6]. Active renin had also been over-expressed in *Escherichia coli *and successfully refolded [7-9]. In order to supply enzyme to support our structure-based design efforts, we developed an efficient system for renin production and purification in amounts sufficient to support routine X-ray crystallography. Herein, we report a simple procedure for renin expression and purification that exploits the affinity of glycosylated renin for Con A. Generally, multi-milligram quantities of material can be prepared from the conditioned medium of HEK cell culture. The active renin protein prepared has been used successfully to support activity assays and to generate high resolution crystal complexes with bound inhibitors.

## Results and discussion

**Figure 1:**
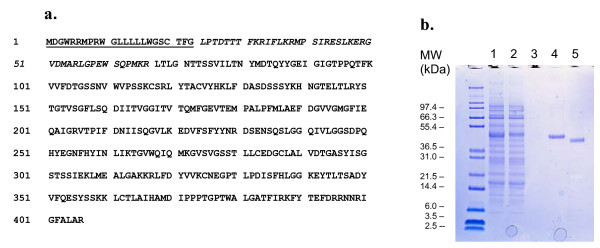
**a. **Amino acid sequence of the recombinant human preprorenin. The underlined 23 residues are the signal sequence (1–23), the residues in italics are propeptide (24–66) and the remaining residues are the mature renin protein (67–406). **b. **SDS-PAGE profile of renin purification. Lane 1, Conditioned medium (load to Con A column); Lane 2, Con A flowthrough; Lane 3, Con A column wash; Lane 4, Eluted prorenin; Lane 5, Prorenin digested with trypsin.

The recombinant human renin gene was transfected into the human kidney cell line, HEK-293. A clonal cell line expressing significant levels of prorenin was prepared by limiting dilution. Analysis of the conditioned media of multiple clones identified a single clone that was scaled and transferred to growth in a hollow fiber bioreactor. This line was subsequently adapted to growth in serum free media. The recombinant human prorenin was constitutively secreted into the medium with concomitant removal of the 23 aminoacid long N-terminal signal peptide (Fig. 1a). Although serum-free medium was used to grow the cells, the small amounts of other proteins (mainly BSA, as analyzed on SDS-PAGE gels) were found as contaminants.

Prorenin has two N-linked glycosylation sites (N_71_TT) and (N_141_GT). The glycosylated prorenin has reasonable affinity for Con A and this interaction was used to purify the protein to high purity in a single step. To our surprise, the major contaminating protein, BSA, did not bind to the Con A resin. The bovine serum had been used in the culture medium initially when the cell line was adapted to the serum-free medium and BSA was a major component. Therefore, the Con A chromatography is more like a step of prorenin enrichment and any major contaminants were removed subsequently with cation exchange chromatography.

After the hollow fiber bioreactor system was set up for the prorenin expression, the conditioned medium was collected, about 30 ml a day, which was pooled and loaded onto the Con A column. For one typical manageable purification experiment, 250–300 ml containing approximately 50 mg proteins was used. The column was washed until the baseline was stabilized. In order to improve the yields, the column was "soaked and then eluted" with the buffer containing D-methyl-mannopyranoside at the low flow rate. Usually, two or three cycles of the soaking/eluting were applied in order to maximize the recovery. The volume of the pooled materials containing mainly prorenin (12–18 mg) was reduced to approximately 30 ml by the Millipore ultrafiltration device, followed by activation and further purification (Fig. 1b). No other detectable endogenous Con A binding proteins were observed by Coomasie stained SDS-PAGE gels.

The Con A material was converted to active renin by digestion with trypsin. Trypsin cleaves after residue Arg_66_. Conditions were optimized using different temperature, time and amounts of trypsin. The final conditions were described in the experimental section and provide mild proteolysis for quantitative conversion of prorenin to active renin (Fig. 1b). Prolonged trypsin treatment did not appear to result in any internal cleavage of the protein, and generally one hour incubation at 4°C with trypsin afforded a complete conversion from prorenin to renin indicating that the propeptide trypsin-cleavage site was accessible.

After digestion, trypsin was removed with trypsin-inhibitor agarose beads. Any other contaminants or misfolded renin proteins were removed with cation exchange chromatography (SP Sepharose Fast Flow, data not shown). Approximately 60–70% of the total proteins were removed at this step. The final step of purification involved with a size exclusion column, resulting in a purified activated renin of >98% purity, as estimated by the reducing SDS-PAGE analysis. The conversion of prorenin to renin by trypsin results in removal of 46 amino acids, approximately 5.2 kDa. Because of heterogeneous nature of glycosylation [10], prorenin and renin migrated on the SDS-PAGE gel as broad bands of around 50 kDa and 42 kDa, respectively (molecular weight of the DNA-deduced polypeptides of prorenin and renin were 42,319 and 37,233, respectively, see Table 1). The proteins were analyzed by the N-terminal sequencing and mass spectrometry for further characterization of the proteolytic activation. The N-terminal sequence analysis confirmed that the first residues of the mature renin were LTLGXT and verified the complete removal of the propeptide (Table 1). The X represents the asparagine residue (N_71_) which is a glycosylation site. Mass spectrometry analysis indicated that glycosylation of renin added from 4 to 7 kDa to the molecular weight, based on the calculated DNA-deduced amino acid sequence. The discrepancy of molecular weights might be related to variations in growth conditions and reagents from batch to batch because of the heterogeneous nature of glycosylation (oligosaccharides varying in the number of sugar residues). The sugar moieties were removable by endoglycosidase H treatment only in partially denatured proteins under conditions of 1% SDS at 37°C overnight (data not shown). In our crystallization studies and biological assays, the sugar moieties were not removed. Typically, 1–3 mg of protein could be obtained from one batch of purification using approximately 250 – 300 ml starting conditioned medium (containing 40–50 mg proteins), depending on cell well-being, growth conditions and other procedural irregularities.

**Table 1:** Summary of physical properties.

Protein	N-terminal sequencing		MALDI-MS	
	
	analyzed	expected	analyzed	expected
prorenin	LPTDTT	LPTDTT	49,580*	42,319
renin	LTLGXT	LTLGNT	41,068*	37,233

*the representative major molecular weight peak from several batch of samples.

**Figure 2:**
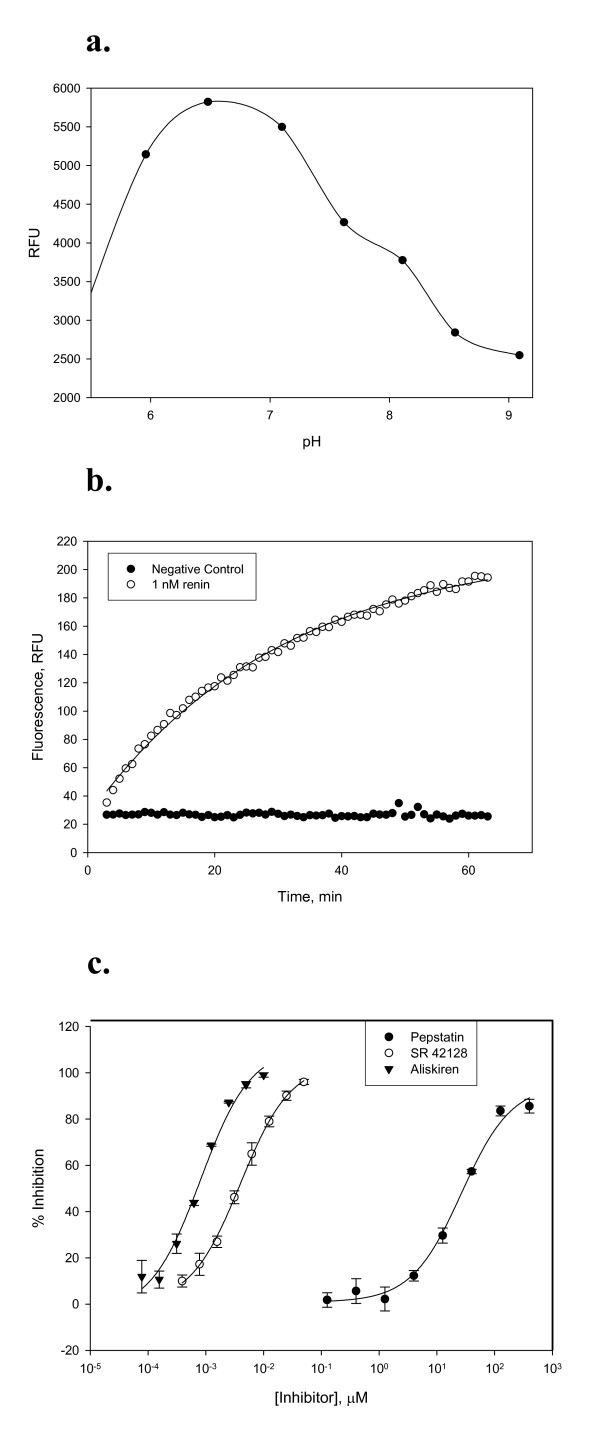
Biochemical characterization of recombinant renin. **a. **The pH rate profile of the hydrolysis of DABCYL-γAbu-Ile-His-Pro-Phe-His-Leu-Val-Ile-His-Thr-EDANS with recombinant human renin. The optimal pH is around pH 6.5 with a poorly defined shoulder at pH 8. **b. **Full progress curve of the cleavage of fluorogenic substrate with recombinant human renin. The enzyme (1 nM) was incubated at 37°C with 1 μM DABCYL-γAbu-Ile-His-Pro-Phe-His-Leu-Val-Ile-His-Thr-EDANS ([S]<<K_M_). **c. **Inhibition of recombinant human renin by standard inhibitors. The percent inhibition is plotted as a function of inhibitor concentration for aliskiren (▼), SR 42128 (○) and pepstatin (●). The line through the data is the least squares fit to a four parameter equation describing inhibition, and the IC_50 _values determined are 0.8 nM (aliskiren), 4 nM (SR 42128) and 31 μM (pepstatin A), respectively.

The activated renin was characterized in a functional assay (Fig. 2). The catalytic activity was measured using the commercially available substrate, DABCYL-γAbu-Ile-His-Pro-Phe-His-Leu-Val-Ile-His-Thr-EDANS, which is based on the angiotensinogen sequence [11]. Velocity of reaction was initially followed by HPLC and exhibited hyperbolic dependence on substrate concentration consistent with Michaelis-Menten kinetics with an apparent K_M _of 3.6 μM (data not shown). This value is similar to the K_M _of 1.5 μM reported in the literature based on the FRET assay [11]. The catalytic efficiency of recombinant rennin was determined using the DABCYL-EDANS substrate. The enzyme had a second order rate constant (k_cat_/K_M_) of 4.7 × 10^5 ^M^-1^s^-1^. This value is close to the published catalytic efficiency of 6.9 × 10^5 ^M^-1^s^-1 ^[6] and 3 × 10^5 ^M^-1^s^-1 ^[12] for the cleavage of angiotensinogen tetradecapeptide by purified human renin [6,12].

Enzymatic activity of the recombinant human renin was measured in a pH-rate profile. Activity was measured between pH 5.4 and 9.1 in 0.5 pH units (Fig. 2a, b). The maximum activity was observed at pH 6.5, similar to purified human renin [2]. The peak and the shoulder of the pH profile were similar to those observed for purified human renin cleaving partially purified human angiotensinogen with the maxima at ~6.5 and 8 [13,14].

Several well characterized inhibitors of human renin were tested with the recombinant enzyme. These inhibitors included commercially available statines and the small molecule inhibitor aliskiren. Pepstatin A (Isovaleryl-Val-Val-Sta-Ala-Sta) is a potent inhibitor of several aspartic proteases including cathepsin E (IC_50 _of 0.2 nM [15]), cathepsin D (IC_50 _of 0.4 nM [16]) and HIV protease (IC_50 _of 250 μM [17]). Human renin is poorly inhibited by pepstatin A (IC_50 _of 15 μM [18] and 36 μM [19]), while porcine renin exhibits sub-micromolar inhibition constants with this compound (IC_50 _of 0.32 μM [18] and 0.58 μM [20]). The recombinant human renin was inhibited by pepstatin A with an IC_50 _of 31 ± 4 μM, similar to literature values (Fig. 2c).

SR 42128 (Isovaleryl-Phe-Nle-Sta-Ala-Sta) is a pepstatin analog that inhibits human plasma renin activity (PRA) with the IC_50 _of 28 nM [21]. The compound is potent against purified recombinant human renin with an IC_50 _of 4.0 ± 0.2 nM (Fig. 2c)

The recombinant renin was also inhibited by aliskiren, a potent human renin inhibitor currently marketed for hypertension as Tekturna [22]. Aliskiren is selective for human renin with a reported IC_50 _of 0.6 nM [23]. In our hands, IC_50 _value for recombinant human renin was 0.81 ± 0.17 nM (Fig. 2c).

**Figure 3:**
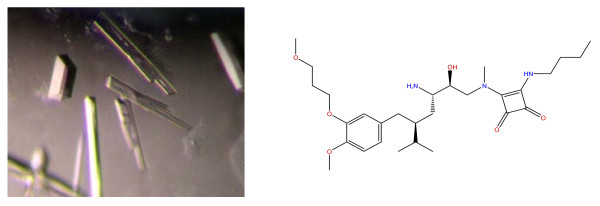
Renin co-crystals complexed with VTP24631.

The recombinant renin is indistinguishable from renin purified from human sources and stable after long term (up to 3 years or more) storage at -80°C. The material is suitable for functional assays and X-ray crystallography. The material had been used to generate >50 renin inhibitor complexes ranging from 1.7Å–2.7Å resolution (unpublished data). A typical experiment is shown with VTP24631 (Fig. 3). VTP24631 is a weak inhibitor of IC_50 _700 nM. Co-crystals of this compound were obtained by the hanging drop method. These crystals diffracted at 2.5Å. This complex can be used for soaking studies with potent inhibitors, which readily displace VTP24631 generating high quality crystals. Details on the structural solutions of VTP24631 and additional structures will be reported separately.

## Conclusion

This present work describes a simple, efficient method for the production and purification of active human renin. Recombinant renin can be generated in milligram quantities via a simple activation/purification sequence. Biochemical properties of the recombinant human renin are indistinguishable from those reported in the literature for purified enzyme. Renin inhibitors were active against the recombinant enzyme with potencies very similar to the inhibition constants measured for purified human renin. The material is highly pure and is suitable for supporting activity assays and structural biology efforts.

## Methods

### Cloning and cell culture

The gene for recombinant human renin protein (accession No. **AAA60363**, 406 amino acid residues) was cloned by PCR from a kidney cDNA library. The polymerase chain reaction conditions were optimized using Elongase polymerase mixture (Invitrogen, Carlsbad, CA) and the following primers: huRenin-fwd-5'-CGT CTA AGC TTG CCA CCA TGG ATG GAT GGA GAA GGA TCG-3' and huRenin-rev-5'-CGG TCA ATT CTA GAC TTC AGC GGG CCA AGG C-3'. The 5'-sense primer introduced a consensus Kozak translation initiation sequence [24] and a *Hind *III restriction site. The 3' antisense primer introduced an *Xba *I restriction site and a stop codon for native protein expression. The PCR product was digested to completion with *Hind *III and *Xba *I (NEB, Beverly, MA) and cloned into the mammalian expression vector pcDNA3.1 (Invitrogen, Carlsbad, CA). The complete renin sequence was confirmed by DNA sequencing performed by BigDye terminator reaction chemistry for sequence analysis on the ABI Prism 377 (Kimmel Center Nucleic Acid Facility, Thomas Jefferson University, Philadelphia, PA). Human embryonic kidney cells, HEK 293 (ATCC, Manassas, VA) were cultured in Eagle's Minimum Essential Medium, supplemented with 10% fetal bovine serum (Hyclone, South Logan, UT), 2 mM L-glutamine, and 100 μg/ml penicillin/streptomycin (Invitrogen, Carlsbad, CA). Transfection experiments with pcDNA3.1-huRenin were conducted using Lipofectamine 2000 (Invitrogen, Carlsbad, CA) according to manufacturer's protocols. Stable transfectants were obtained by selection in media supplemented with Geneticin (800 μg/ml), and single clones were obtained by limiting dilution. A single clone was scaled up and introduced into a hollow fiber bioreactor using a medium sized cartridge with a 5 kD molecular weight cut-off (Fibercell Systems Inc, Frederick, MD) to trap the secreted enzyme in the extra-fiber space. Once the cell line was established in the bioreactor it was adapted incrementally to a serum-free media for HEK-293, SFM4HEK293 (Hyclone, South Logan, UT) which would aid purification of the protein. Protein was harvested from the extra-fiber space daily and the cell line was maintained in the bioreactor for over 6 months with no observable decrease in protein production.

### Protein purification

The clarified conditioned medium (typically 250 – 300 ml), was loaded by gravity at 4°C to a 30 ml Concanavalin A (Con A) column (Pharmacia/GE Healthcare, Piscataway, NJ), which had been equilibrated with Buffer A (25 mM Tris-HCl, pH 7.5, 300 mM NaCl, 0.1 mM CaCl_2 _and 0.1 mM MnCl_2_). The column was then connected to the FPLC system (GE Pharmacia/GE Healthcare, Piscataway, NJ) and washed with 5 volumes of Buffer A or until the baseline was stabilized. The prorenin protein was eluted in Buffer A supplemented with 150 mM D-methyl-mannopyranoside (Sigma-aldrich, St. Louis, MO) at slow flow rate (0.5 ml/min) in a "soak/elute" fashion. The propeptide was removed by trypsin digestion (1:100 by weight, trypsin:prorenin) at 4°C for 60 minutes. The reaction was quenched by the addition of trypsin-inhibitor (from soybean) beads (T0637, Sigma-aldrich, St. Louis, MO). The activated renin was dialyzed overnight at 4°C against Buffer B (10 mM sodium acetate, pH 5, and 10 mM NaCl). This solution was chromatographed on the 50 ml SP Sepharose Fast Flow column (Pharmacia/GE Healthcare, Piscataway, NJ) using a NaCl gradient (10 mM – 500 mM). Homogeneous renin was obtained after the gel filtration column Superdex 75 HR 10/30 (Pharmacia/GE Healthcare, Piscataway, NJ). The purified renin in a buffer containing 20 mM Tris-HCl, pH 7.0 was concentrated to 5 mg/ml, by ultrafiltration using an Amicon stirred cell (Millipore, MA) with a membrane of 10,000 MWCO (YM10), and stored at a -80°C freezer until future use. No deglycosylation was performed.

Protein samples were analyzed by matrix-assisted laser desorption ionization mass spectrometry (MALDI-MS) and by Edman's N-terminal sequencing (the Wistar Institute, University of Pennsylvania, Philadelphia, PA).

### SDS-PAGE and protein assays

The protein samples were analyzed using precast NuPage Novex 4–12% Bis-Tris gels with MES running buffer (Invitrogen, MD). Proteins on the gel became visible after staining with 0.01% Coomassie Blue R in a solution containing 10% glacial acid and 20% methanol. Protein concentrations were measured using Bio-Rad Assay Kit (a Bradford-based dye binding method [25]), with bovine serum albumin (BSA) as standard.

### Functional assay

The fluorescence resonance energy transfer (FRET) assay for renin activity was performed essentially as described in literature [11]. Recombinant human renin (0.3 nM) was incubated with 1 μM FRET substrate DABCYL-γAbu-Ile-His-Pro-Phe-His-Leu-Val-Ile-His-Thr-EDANS (AnaSpec, San Jose, CA) in the 50 mM BES buffer, pH 7.0 containing 0.5 mg/ml BSA, 150 mM NaCl and 2% DMSO. Incubation was performed at room temperature in a final volume of 200 μl in white opaque 96-well optiplates (PerkinElmer, Wellesley, MA). Fluorescence (λ_ex_= 336 nm, λ_em_= 495 nm) was measured repeatedly over extended period of time (1–2 hrs) on either Victor V or Fusion plate reader (PerkinElmer, Wellesley, MA), and the intensity of fluorescence was regressed against reaction time to derive velocities. The reaction rates were used for calculating percent inhibition values using uninhibited renin as a positive control and either no enzyme or renin inactivated with 2 μM SR 42128 (Isovaleryl-Phe-Nle-Sta-Ala-Sta) (Bachem, King of Prussia, PA) as a negative control. IC_50 _values were calculated by fitting the percent inhibition values *vs. *inhibitor concentration into a four-parametric model using XLFit software (IDBS, Guildford, UK).

### Sample preparation and crystallization

The hanging-drop, vapor-diffusion method was used for renin crystallization. Renin was first mixed with 2 mM VTP24631 and incubated on ice for 30 minutes. For each hanging drop, 1 ul of the protein solution was combined with 0.6 ul of precipitant (0.1 M Tris-HCl, pH 7–8.2, 0.2 mM (NH_4_)_2_SO_4 _and 18–26% PEG 3550). Crystals were grown at 4°C for 5–7 days and streak seeding was required for crystals to reach their maximal sizes (200–500 μm).

## Abbreviations

BES: N, N-bis(2-hydroxyethyl)-2-aminoethanesulfonic acid; BSA: bovine serum albumin.

## Authors' contributions

ZW designed and carried out the purification and crystallization studies, and drafted the manuscript. MGC and BBS carried out molecular cloning, cell culture work and contributed to the manuscript. YB conducted the biological assays and contributed to the manuscript. GMMcG conceived the study and participated in its design and coordination and revised the manuscript. All authors read and approved the final manuscript.
